# Evaluation of Registered Visually Disabled Individuals in a District of West Bengal, India

**DOI:** 10.4103/0970-0218.42057

**Published:** 2008-07

**Authors:** Sambuddha Ghosh, Subhalakshmi Mukhopadhyay, Krishnendu Sarkar, Manas Bandyopadhyay, Dipankar Maji, Gautam Bhaduri

**Affiliations:** Regional Institute of Ophthalmology, Kolkata, India; 1 Department of Pathology, Midnapore Medical College, West Bengal, India; 2 National Institute of Epidemiology, Chennai, India

**Keywords:** Certification, congenital abnormality, visually impaired individuals

## Abstract

**Objective:**

To identify the sociodemographic characteristics, degree and cause of visual disability among certified visually disabled individuals in a rural district of West Bengal, India and to identify possible lacunae, if any, in the existing certification system.

**Materials and Methods:**

A cross-sectional study by secondary data analysis of medical records of 155 visually disabled individuals and their 310 eyes. Demographical features, diagnosis, percentage of visual disability and work activity status of each individual were analyzed.

**Results:**

One hundred and thirty one (84.52%) individuals had 100% disability. The number of males was significantly higher than that of females. Fifty eight (37.42%) individuals were below 21 years of age. Phthisis bulbi was the most common cause followed by microphthalmos. Further, 81.29% patients had the same lesion bilaterally.

**Conclusion:**

Patients with higher grades of disability have attended certification boards. A large number of disabled individuals comprised children and young adults. Male gender bias demands concern.

Certification for blindness or partial sight is the process by which social services for the visually disabled are coordinated. Registration as blind or partially sighted in India is voluntary and is performed by certification by a duly constituted board that includes ophthalmologists. Defining disability is difficult to accommodate the expectations of all disabled groups. According to a guideline by the Ministry of social justice and empowerment of Government of India, the minimum degree of disability should be 40% for an individual to be eligible for any concessions or benefit.(1) The National Sample Survey Organization (NSSO)** that conducted a survey of individuals with disabilities in 1981, 1991 and 2002 in India, considered disability as ‘Any restriction or lack of abilities to perform an activity in the manner or within the range considered normal for a human being’. The 58^th^ round data from the NSSO survey reveals that of all disabled individuals in India, 10.88% were blind and 4.39% were having low vision.(2) Prevention of visual impairment is an international priority, and its planning requires contemporary data regarding incidence and causes based on which priorities can be identified. However, under-registration of the blind is a global problem.(3,4) It is also important to know whether the present certification system fulfils the need of all sections of the society. No such data is available from the state of West Bengal (Medline search). The present study was designed to conduct a secondary data analysis of disabled individuals belonging to South 24-Parganas district, West Bengal, India, who were certified as visually disabled by duly constituted medical boards. The district has predominantly rural population, including people living in remote Sunderban area. The objectives of our study were to analyze the medically certified visually disabled individuals based on sociodemographic characteristics, degree of disability and the cause of visual disability as well as to identify possible lacunae, if any, in the existing certification system.

## Materials and Methods

Patients with visual disability of 40% or above were included in this study. The percentage of disability was calculated based on the guidelines for the evaluation of various disabilities and procedure for certification [Table 1].(1) Sample size was calculated considering the proportion of females among the visually impaired to be 57% (according to the NSSO survey(2)), considering the confidence interval as 95%, confidence coefficient as 8% of the sample proportion and 5% margin for cases with incomplete records for a simple random sample. There were two designated centers in South 24-Parganas district for the disability assessment for certification. Patient data were collected from records in the disability register of these centers and analyzed. We included all 155 cases (310 eyes) certified by the medical board at those centers over a three-year period. Patients attending the board were examined in the outpatient department. Diagnosis was based on medical history, clinical examination and special investigation such as tonometry and automated perimetry as and when necessary. In our study, visual disability was defined as not less than 40% disability as certified by a duly constituted medical board. The variables of interest for this analysis were the following: age, gender, religion, percentage of disability and causative factor in each eye and work activity status of the disabled individual. Statistical analyses were performed by using the Epi-Info version 6 by summarizing the data in terms of mean, median, mode and proportion. Significance of difference of proportions was determined with chi square test.

**Table 1:** Categories of visual disability

Best corrected visual acuity in the better eye	Best corrected visual acuity in the worse eye	Percentage of impairment
6/18–6/36	6/60 to nil	40
6/60–4/60 or field of vision 10°-20°	3/60 to nil	75
3/60 to 1 /60 or field of vision 10°	Finger count at 1 ft. to nil	100
F.C. at 1 ft. to nil or field of vision 10°	Finger count at 1 ft. to nil or field of vision 10°	100

## Results

The study was conducted on 155 individuals and their 310 eyes. Of these individuals, 109 (70.3%; 95% CI: 63.1, 77.5) were male (M) and 46 (29.7%; 95% CI: 36.9, 22.5) were female (F), the M:F ratio being 2.37:1. Among the study population 74 (47.7%; 95% CI: 55.6, 39.8) were Hindu and 81 (52.3%; 95%CI: 56.3, 48.2) were Muslim by religion. One hundred and thirty one (84.5%; 95% CI: 90.2, 78.8) individuals were 100% visually disabled, 14 (9.0%) had 75% visual disability and only 10 (6.4%) had 40% visual disability [Table 2]. The M:F ratio was 2.04:1, 13:1 and 2:1 among the 100%, 75% and 40% disability groups, respectively. Forty three (93.5%) of the females and 88 (80.7%) of the males were 100% disabled [Table 2]. The age range was 5–74 years, with the mean age of 29.5 years (95% CI: 27.1, 31.9), median of 26 years and mode of 40 years. Ninety four (60.6%; 95% CI: 68.3, 52.9) patients were in the working age group (age range, 21–60 years); further, only 3 (1.93%) individuals were above 60 years of age and 58 (37.4%) individuals were below the age of 21 years [Table 2]. There was no significant difference in the distribution of males and females above or below the median age (*P*= 0.06). There was a significant difference between the proportion of individuals with 100% disability and those with below 100% disability (*P*= 0). The correlation coefficient between the age and degree of disability was 0.12. Of all disabled individuals, 69.03% were unemployed, 80.44% among females and 64.22% among males [Table 3]. Among the visually disabled, phthisis bulbi was the most common cause of disability, being present in 17.74% (95% CI: 21.99, 13.49) eyes; microphthalmos was the second most common cause closely followed by leucoma [Table 4]. In 38.71% eyes, disability was due to some congenital or developmental defects. In the cases with congenital or developmental defects in both eyes, the median age was 18 years. Of the 11 patients with retinitis pigmentosa, only 3 (27.27%) were below 21 years of age. All 9 patients with congenital cataract were aged 10 years or more.

**Table 2:** Distribution of visually disabled individuals according to the age, gender and percentage

Age in years	100%		75%		40%		Total cases (n = 155)%
				
	M	F	M	F	M	F	
>60	2	0	1	0	0	0	(3) 1.93
51–60	7	1	1	0	0	0	(9) 5.81
41–50	18	6	1	0	1	0	(26) 16.77
31–40	17	5	2	0	1	0	(25) 16.13
21–30	18	12	2	1	1	0	(34) 21.94
11–20	18	14	4	0	5	2	(43) 27.74
5–11	8	5	0	2	0	0	(15) 9.68
Total cases	88	43	13	1	8	2	(155) 100

**Table 3:** Work activity status of disabled individuals

Work activity status	Male (n = 109)%	Female (n = 46)%	Total (n = 155)%
Self-employed in agriculture	(16) 14.68	(0) 0	(16) 10.33
Self-employed in nonagriculture	(1) 0.91	(0) 0	(1) 0.65
Regular employee	(1) 0.91	(0) 0	(1) 0.65
Casual employee	(3) 2.75	(3) 6.52	(6) 3.87
Unemployed	(70) 64.22	(37) 80.44	(107) 69.03
Attending educational institution	(6) 5.51	(0) 0	(6) 3.87
Attending domestic work	(6) 5.51	(3) 6.52	(9) 5.8
Begging	(6) 5.51	(3) 6.52	(9) 5.8

**Table 4:** Distribution of eyes with disability according to the causative factor

Cause	Both eyes	One eye	Total eyes
Phthisis bulbi	34	21	55 (17.74)
Microphthalmos	38	3	41 (13.23)
Central leucoma	26	12	38 (12.26)
Retinitis pigmentosa	22	0	22 (7.10)
Optic atrophy	22	0	22 (7.10)
Glaucoma	18	0	18 (5.81)
Congenital cataract	16	1	17 (5.48)
Anterior staphyloma	10	5	15 (4.84)
Macular dystrophy	14	0	14 (4.52)
Myopic degeneration	10	2	12 (3.87)
Coloboma	8	2	10 (3.23)
Retinal detachment	8	2	10 (3.23)
Anophthalmos	6	3	9 (2.90)
Amblyopia	4	3	7 (2.26)
Corneal dystrophy	4	1	5 (1.61)
Central choroiditis	4	1	5 (1.61)
Occlusio pupillae	2	1	3 (0.97)
Corneal degeneration	2	0	2 (0.64)
Diabetic retinopathy	2	0	2 (0.64)
Buphthalmos	2	0	2 (0.64)
Retinoblastoma	0	1	1 (0.32)
Total	252	58	310 (100)

Figures in parenthesis are in percentages

## Discussion

Our observations conform to those of previous studies at different geographical settings. In one cross-sectional survey of certification at the Moorfields Eye Hospital in the United Kingdom (UK), 51% of the patients identified as eligible for registration as disabled did not have a certificate.(3) In another study in the UK, of the 146 eligible patients, 45% were unregistered.(4) Of them, 18 were blind and 47 were partially sighted. These findings suggest that under-registration of eligible blind and partially sighted individuals is a global problem. In India, No study result with regard to this problem has been available (Medline search). It was evident from our study that the number of males attending the medical board to obtain the disability certification was significantly higher than that of the females. In the 58^th^ round of the NSSO survey,(2) nearly 54% of the total visual impaired individuals were females and the remaining 46% were males, depicting a female gender bias. This trend persisted in the rural West Bengal with a prevalence rate of 251 female and 189 male per 100000 populations.(2) However, the NSSO survey has defined visual disability differently as has been described in our series. As discussed in our study, the existing certification system was institution-based and hence a problem of access for the females might occur due to the social and economic obstacles for certification. In our study, young patients were in a significant majority compared to the elderly. This suggests that the driving force to attend the board for disability certification was more among the younger individuals. This was probably due to the presence of certain benefits associated with disability certification such as employment, education and conveyance, which are more likely to serve the purpose of young individual than the elderly. Similar observations were made in one study where noncertification was found more common in patients of 65 years or more than those under 65 with a trend of increasing odds with increasing age.(3) In our study, patients with 100% disability were in a majority compared to patients with disability of lower grades. Similar finding was noted in a study in the UK where a partially sighted ophthalmic outpatient is estimated to be three times more likely to be noncertified than a blind patient with similar diagnosis.(3) Unemployment in our series was consistent with the findings of the NSSO survey,(2) which observed that 80% of the blind individuals in the rural areas are without any source of income and the remaining are mostly employed in low-profile jobs and only 3% of the blind individuals are regular employees, the rest being either casual workers or attending domestic chores. The low vision individuals also depict similar situation. This is not difficult to imagine the huge obstacle a visually disabled unemployed elderly may have to face to reach the medical board for a disability certificate. Benefits of certification do not appear enough to motivate elderly patients to attend board in our study. Apart from geographical distance, cost of conveyance, loss of wage and procedural complexities may all act as prohibitory factors. Outreach camps may help elderly and females. In our study, phthisis bulbi was the most common cause of disability. Early intervention on certain occasions can prevent this disastrous condition. Microphthalmos was the second most common cause ahead of corneal opacity. Congenital and developmental anomalies were present in as high as 38.71% eyes. This finding may be explained by the fact that we studied predominantly young patients. However, factors such as consanguinity and congenital rubella syndrome are associated with such developmental disorders in India.(5,6) Congenital abnormalities worldwide (microphthalmos, anophthalmos and coloboma) account for severe visual impairment and blindness in 18% and 25.8% of blind school children in South and North India, respectively.(7,8)

One limitation of this study was that it was dependent on the quality of information recorded and could not be verified. Another limitation was that since we used the hospital data, we had no specific population denominator; therefore, the rates could not be calculated and we depended only on the number of cases.
